# Not All Structure and Dynamics Are Equal

**DOI:** 10.3390/e23091226

**Published:** 2021-09-18

**Authors:** Garrett Mindt

**Affiliations:** Elizabeth R Koch Research Fellow, Tiny Blue Dot Foundation for Consciousness Studies, Department of Psychiatry, University of Wisconsin–Madison, Madison, WI 53719, USA; mindt@wisc.edu

**Keywords:** consciousness, physicalism, structure and dynamics, hard problem, complexity, Integrated Information Theory, intrinsic, extrinsic, meaning

## Abstract

The hard problem of consciousness has been a perennially vexing issue for the study of consciousness, particularly in giving a scientific and naturalized account of phenomenal experience. At the heart of the hard problem is an often-overlooked argument, which is at the core of the hard problem, and that is the structure and dynamics (S&D) argument. In this essay, I will argue that we have good reason to suspect that the S&D argument given by David Chalmers rests on a limited conception of S&D properties, what in this essay I’m calling extrinsic structure and dynamics. I argue that if we take recent insights from the complexity sciences and from recent developments in Integrated Information Theory (IIT) of Consciousness, that we get a more nuanced picture of S&D, specifically, a class of properties I’m calling intrinsic structure and dynamics. This I think opens the door to a broader class of properties with which we might naturally and scientifically explain phenomenal experience, as well as the relationship between syntactic, semantic, and intrinsic notions of information. I argue that Chalmers’ characterization of structure and dynamics in his S&D argument paints them with too broad a brush and fails to account for important nuances, especially when considering accounting for a system’s intrinsic properties. Ultimately, my hope is to vindicate a certain species of explanation from the S&D argument, and by extension dissolve the hard problem of consciousness at its core, by showing that not all structure and dynamics are equal.

## 1. Introduction

The structure and dynamics (S&D) argument from Chalmers [1] is taken as a backbone of the hard problem of consciousness [2,3]. The hard problem of consciousness is the problem of why there is any experience associated with certain physical processes. Here, we are taking physical processes to be those describable by the tools of the physical sciences, which Chalmers characterizes as structural and dynamical features. Structure here is meant to refer to the spatial/temporal and formal features of some phenomenon and dynamics the causal evolution and capacities of some given phenomenon. This, for the most part, has been accepted as a plausible description of what consists of the “physical” (cf. Torin Alter [4] for a thorough treatment of the structure and dynamics argument). Largely this is due to Russell’s description of what physics is in the business of describing, namely the spatial/temporal/nomic and mathematico-causal description of the world [5]. The disagreement comes in the gap it purportedly shows between the physical and the mental. These gaps are centered around a set of closely related arguments aimed at showing that physical facts do not entail phenomenal facts, and thus there is an epistemic gap between the physical and phenomenal [1,6,7,8]. Some then go on to argue that this epistemic gap entails a metaphysical/ontological gap, and if this is so, then physicalism must be false. Herein, I am not so much concerned with showing that physicalism as a metaphysical thesis is false or argue that anti-physicalism is preferred; rather I want to examine the structure and dynamics argument itself and show that it may not offer a nuanced enough description of structure and dynamics. This is not to show that physicalism is correct, but rather that structure and dynamics, as Chalmers has described, are not the whole story.

My considerations of the structure and dynamics argument come from the perspective of Integrated Information Theory (IIT) of consciousness [9,10,11,12,13,14,15], which claims there is an explanatory identity between properties of experience and properties of the cause–effect structure (CES). Recently, I’ve argued [16] that IIT is susceptible to the structure and dynamics argument, and thus does not solve the hard problem, aside from falling victim to other concerns regarding physicalism. In this essay, I will be turning my sights on this line of argument and will be showing why the structure and dynamics argument does not apply to IIT, if we develop a more nuanced understanding of structure and dynamics. My hope is that the present work will go some way in showing why the sort of causal-structuralist explanation of consciousness IIT gives is heading in the right direction when it comes to both explaining phenomenal experience naturalistically, as well as one that overcomes the hard problem.

I will begin in Section 2 by giving a brief overview of physicalism and the structure and dynamics argument. In Section 3, I will give a very brief overview of the relationship between different notions of information, to better highlight the issue in information-theoretic terms. In Section 4, I will explore recent work done in the complexity sciences on giving a naturalized definition of semantic information, one that is substrate independent. By substrate independent, I just mean that the nature of that information is not reliant on it having any particular kind of realization base, whether that be the mushy and pulsing throb of organic matter, the silica of a computer chip, or any other kind of substrate base one might conceive. Section 5 will look at IIT and its relationship to a more nuanced understanding of structure and dynamics as well as its position in approaching the hard problem of consciousness. Section 6 will summarize what the preceding discussions in Section 2, Section 3, Section 4 and Section 5 have revealed and argue that Chalmers’ [1] S&D argument fails once we see that not all structure and dynamics are equal. Section 7 concludes with a brief discussion of what I think that the implications are for the metaphysics of consciousness more generally.

## 2. What Is Physicalism? What Are Structure and Dynamics?

Physicalism although often considered the dominant metaphysical position, is a notoriously tricky beast to tackle in terms of its actual definition (I’ll be using physicalism and materialism interchangeably in this essay to ease exposition). I will, however, begin this section with a short discussion on what we mean when we say some thing or process is *physical* and what we mean when we ascribe to the metaphysical position called *physicalism*.

So, what is physical? And what is physicalism? To begin, let us look at what it means for some thing or process to be *physical*. Stoljar [17] offers an apt definition of what it means for something to be physical (and is the same conception which Chalmers has in mind in his arguments as well). Stoljar calls this the *theory-based conception of physical* and defines it as:

**The theory-based conception (TBC)**: “A property is physical *iff* it is the sort of property that physical theory tells us about”.

[17]

So, we can consider that which is physical to be anything that falls under the purview of those objects/properties/processes posited as part of some physical theory. For example, mass is posited as a property of objects according to physics and would thus qualify as a physical property according to the TBC. We can now move to defining what the metaphysical view of physicalism would thus amount. Lewis [18] offers a definition of physicalism (after much back and forth on possible definitions).

**Physicalism/Materialism**: “Among worlds where no natural properties alien to our world are instantiated, no two differ without differing physically; any two such worlds that are exactly alike physically are duplicates”.

[18]

Another way to put this is that there is nothing over and above the physical, there is no difference without physical difference, etc. That being said, Chalmers [2,3] worries were that it does not seem to be the case that phenomenal experience fits into such a world view, since it is not clear how the felt aspect of experience, or the what-it is-likeness of experience, is entailed by such physical properties. Another way to put it is that it is not clear that the existence of certain physical properties entails the existence of identical phenomenal properties. This is sometimes framed in terms of the conceivability argument [1,8], the knowledge argument [6], and the explanatory gap argument [7].

That being said, there are some interesting processes we might speak of in the context of the physical, namely certain structural and dynamical processes/properties of systems. Chalmers defines structural and dynamical properties as:

“First: a microphysical description of the world specifies a distribution of particles, fields, and waves in space and time. These basic systems are characterized by their spatiotemporal properties, and properties such as mass, charge, and quantum wavefunction state. These latter properties are ultimately defined in terms of spaces of states that have a certain abstract structure (e.g., the space of continuously varying real quantities, or of Hilbert space states), such that the states play a certain causal role with respect to other states. We can subsume spatiotemporal descriptions and descriptions in terms of properties in these formal spaces under the rubric of structural descriptions. The state of these systems can change over time in accord with dynamic principles defined over the relevant properties. This result is a description of the world in terms of its underlying spatiotemporal and formal structure, and dynamic evolution over this structure”.

[1]

As an example, take my current spatiotemporal location at the moment of the writing of this sentence, that being my living room, in my condo, in Madison, Wisconsin in 2021. We can provide further specification in further and further detail—the sofa in my living room on 19 March 2021 at 2:44 pm, or down to the excruciating detail of all the components which compose my body, down to the most abstract and inconsequential details of my atomic structure. Mapping any of these features would be a structural description (of a certain variety) of myself. This, along with those formal descriptions of the properties associated with those spatiotemporal entities and the various nomic relations which hold between them encompass what can be called the “structural” properties of those systems/entities. Aside from this, I have interesting dynamical features, not merely the structural features describing the state of all my atoms in my body. I not only occupy a unique position in spacetime, but also carry a number of causal capacities and my states evolve through time dynamically, governed by certain dynamic principles. For instance, my location in spacetime is accompanied by my ability to retrieve a beer from the fridge and to type an essay on my laptop (I have limited dynamical capacity for much else), and these changes in states evolve over time. There is thus a kind of symphony of structural and dynamical features of myself that describe my body and its interaction with the world throughout my entire existence. Furthermore, these features of my body (the system in question) are amenable to third-person observation. Someone could put me in some futuristic machine that details every component of my body in excruciating detail and give a full description of these structural and dynamical features. One could call all those details and explanation of these processes the *physical*, though I think a more apt description would be the *extrinsic features* of a system, allowing one to both stay neutral on the battle of physical vs. non-physical, and to rather get at the more interesting questions concerning processes and interaction (see Section 6 for a more detailed explanation of this).

The context in which this essay and that of the S&D argument originally was targeted warrants comment on what I as well as Chalmers mean when we speak of consciousness or experience. By consciousness, in this essay, I will be adopting the phenomenal sense of the word, as in a system can be said to possess consciousness or undergo phenomenal experience when there is *something it is like* to be that system, some kind of felt experience [19]. The question is, can S&D of the kind Chalmers describes explain the presence of phenomenal experience; that is do such explanations generate a plausible account of why and how certain systems have phenomenal experience rather than others? Chalmers concludes they do not, since essentially they only give one relational and dispositional description of the world (as Stoljar [20] characterizes Chalmers’ S&D argument) and, as he argues in Chalmers [2], this is insufficient to capture phenomenology and thus fails to account for the Hard Problem.

There are actually two questions of relevance in this discussion: (1) Can S&D of the kind Chalmers describe capture phenomenal experience and explain consciousness? Additionally, much more importantly for the present work, (2) is there only the kind of extrinsic S&D which Chalmers describes in his argument? The focus of this essay is on the second question and note that the veracity of an answer to the first question only concerns S&D as Chalmers describes it (what I am calling *extrinsic S&D*) and does not apply to anything outside his description. Section 3 will be focused on motivating that Chalmers S&D argument paints S&D features of a system with too broad a brush and misses important nuances in certain structural and dynamical features of a system. Before we do this though, it will be useful to go over some broad-brush strokes on the various kinds of information that will be discussed in this essay. Since the species of explanation we will be investigating are information-theoretic, it will be helpful in understanding the various goals of understanding the connection between various types of information, specifically between syntactic information, semantic information, and intrinsic information.

## 3. From Syntax, to Semantics, towards Intrinsic Information

Some space should be given to better lay the groundwork for what an information-theoretic explanation of consciousness should be striving towards, if we are to better appreciate the insights gleaned from complexity science and IIT with regard to the structure and dynamics argument. I broadly see the project of giving an information-theoretic explanation of consciousness, one which overcomes the hard problem and S&D argument, as giving an explanation of how syntax relates to semantics and how these relate to intrinsic information. Each of these different notions of information will be given an explanation within this section to better elucidate how a more nuanced understanding of structure and dynamics might shed light on possible explanations of how phenomenal experience comes about in certain naturally occurring complex systems.

Shannon’s [21] original entropy measure of the engineering behind communication channels was explicitly concerned with the operation and formal properties of how messages are sent from a signal and receiver, specifically the channel conditions through which messages were sent, and not with the meaning associated with those messages. Given some arbitrary alphabet and certain conditions of the communication channel, Shannon’s notion gave a universal formal description of how information is sent from a sender to a receiver. Essentially, Shannon separated the question of how messages have any meaning and set this to one side (as pointed out by Bar-Hillel and Carnap [22] in their criticism of Shannon’s notion of information). Faulting Shannon for this though would be a mistake, since his goal was not to explain how meaning is transmitted via some communication channel, but rather, how a communication channel operates and functions universally, in such a way that any possible message might be transmitted via some communication channel. In this sense, Shannon’s purely syntactic or extensive notion of information, is perfectly fit for form. However, what about in the instance when we are not merely interested in whether a system possesses, transmits, or receives information, but how the meaning associated with those messages occurs, that is, what the semantic content of the message is and is there a formal way of expressing the relationship between syntactic information and semantic information?

There have been attempts over the decades since Shannon’s landmark entropic notion of information. Bar-Hillel and Carnap [22] attempted to account for the semantic content by the propositional content of the messages being transmitted, though failed to give a full account. Dretske [23] attempted to give a more unified picture of information through understanding knowledge and belief as functions of information-theoretic relationships. Of course, with the drawback that it required a sort of backdoored notion of innate content to get the whole picture off the ground. More recently, Floridi [24] has given what he calls a general definition of information (GDI) in which he outlines that information (in the semantic sense) is well-formed, meaningful, and truthful data. From the side of physics [25] and complexity sciences [26] a slightly different approach is taken to explaining how you move from pure syntactic relationships to semantic content. In Section 3, this will be gone over in more detail, but roughly the project consists of understanding semantics as a function of a system’s ability to causally maintain its existence over time. Those syntactic relationships which exist between an organism/system and its environment have value (i.e., have semantic content) if the syntactic information has the result of helping the system causally maintain its existence over time. This story of how meaning is generated has the upshot of fitting in nicely with our evolutionary story of how organisms such as ourselves came about, while also having a clear formal explanation. Of course, this work is in its nascent stages but shows promise in bridging the seeming divide between syntax and semantics. This explanation of how to delineate between syntax and semantics will be gone over in more detail in Section 4, this section is merely meant to give broad brush strokes on what kinds of information we are concerned with in our explanations of consciousness via structure and dynamics.

The next step is to go from syntax and semantics to the intrinsic or internal perspective of that information. So not only that the information has meaning but what it is like for some organism/system to have that semantic information. Since the question is not merely whether some organism/system has semantic states, i.e., say mental states with some sort of meaningful content associated with them; but rather that those mental states fall under the watchful gaze of that same organism/system, that is to say that semantic content is experienced. This notion of intrinsic information finds its clearest and most powerful exposition in the work of those developing IIT, as it is clearly stated: “...we can think of the integrated information Φ^max^ as a measure of the intrinsic phenomenal capacity of the [cause–effect structure] specified by the PSC [physical substrate of consciousness]. By contrast, Shannon information can be used to measure the extrinsic access capacity of a channel that runs from a subset of elements of the PSC to Broca’s area and from there to motor neurons that ultimately convey the report.” [14]. The quote has been modified to reflect IIT’s most recent exposition changing “conceptual structure” to [cause–effect structure] in order to make the exposition clearer and in-line with the most recent conceptual developments of IIT. The same has been done with the change of “concept” to [distinction] throughout the quotes of IIT.

IIT’s aim is to capture the information density of the structure specified by the physical substrate, i.e., the information intrinsic to the system despite anything external to it. It does not aim to characterize the information which the system receives, say from the external world (via the various sense modalities), or the information it transmits to the world (say through verbal report or communication). That is not to say such information is not relevant at some stage of explanation but merely that it is not one of the essential features to explain when considering how a system is conscious from the intrinsic perspective. More recently, Barbosa et al. [27] and Barbosa et al. [28] have developed a unique distance measure for intrinsic information, derived from the postulates of Integrated Information Theory (IIT) of consciousness (a short introduction to IIT will be given in Section 5). The basic idea is to capture not just the amount/value of information in the system, but the quality of that information in spite of a sender/receiver, i.e., what information is held intrinsically for the system. Why might this be relevant in the current context? If the ultimate goal is to understand how structural and dynamical explanations can give one a full naturalized account of consciousness our best path forward is to understand how syntactic information can result in semantic information, and finally what information constitutes consciousness, is held intrinsically for the system, the meaning for the system from the intrinsic perspective of that system.

## 4. Complexity Sciences, Meaning, and Intrinsic Structure and Dynamics

Recently, Luis Favela [29] has argued that IIT is best understood under the rubric of complexity sciences more generally, as he phrases it “I claim that IIT is a complexity science approach to consciousness. Even though there is no single ‘complexity science’, there are typical concepts, methods, and theories that fall under that heading” [29]. Favela argues that IIT and complexity sciences share an important feature which makes them natural bedfellows, namely, a focus on *interaction dominance* rather than *component dominance*. Favela takes this notion from Bechtel and Richardson [30], whose aim was to understand the extent to which mechanistic explanation fit the models with which scientists were concerned, and in what ways they did not. Mechanistic explanations are appropriate and in fact the best way to go in explaining say some biological phenomenon when that phenomenon entails systems that are component dominant, meaning they are about the individual parts and mechanisms that compose a system and not about the interactions between those parts and/or mechanisms. Interaction dominant systems are concerned with the opposite, when the behavior of a system is not dependent on the parts or mechanisms themselves but their interactions with one another. A common example that illustrates this, used by Favela, is the difference between a toilet and a locust swarm. A toilet functions as a result of the organization of its parts, and this function is decomposable to those components. An explanation of the behavior of such a system is thus analyzable mechanistically. As for the locust swarm the individual components themselves (the individual locusts) are not responsible for the swarm’s behavior or movements, but rather it is the interaction between the individual components. A way to think of this is that in the case of the swarm, the global dynamics of the system are what is important to the system rather than the individual components dynamics, and with the toilet the individual components dynamics are what is important and not the global dynamics of the system.

Given the historical trajectory of IIT, it is clear that capturing the complex interactions of brain-wide and neural interactions was at the very foundations even before IIT as a theory was developed in earnest starting with Tononi’s work on capturing the effective information and neuronal complexity of neuronal interactions [31,32,33]. Additionally, then subsequent work on information integration being a key component in understanding consciousness [34,35]. These earlier developments led to a more full-fledged form of Integrated Information Theory [13,36], or what might be called IIT 1.0. Even given the connection of the earlier work with the latter work the relationship between IIT and complexity sciences is both important in some respects, and in others superficial. Since IIT goes the necessary step further than complexity sciences in understanding the intrinsic and internal S&D as it relates to consciousness (this will be fully explained in Section 6). This will be more relevant in the following section, but it is worth noting, so as not to misidentify IIT as capturing just extrinsic complexity.

In a recent essay, Kolchinksy and Wolpert [26] explore how to give a naturalized definition and explanation of semantic information drawing from work in non-equilibrium statistical physics and experiments with autonomous agency, all under the framework of complex systems theory. The notion of semantic information they are working with is as follows:

**Semantic information**: the information that a physical system has about its environment that is causally necessary for the system to maintain its own existence over time.

[26]

The question is how does semantic information naturally arise as the result of certain interactions? We want to give a naturalistic explanation about how semantic information can result from what appears to be non-meaningful physical interactions, and thus one must produce a story about how that occurs. Someone may object here that the way in which I am using semantic information deviates from the norm we would usually expect. That person would be correct to point this out, and that is precisely the point of why I want to introduce this somewhat non-standard notion of semantics. We have seemed to make little progress in bridging the gap we are concerned with in our understanding of nature by investigating semantics propositionally (such as Bar-Hillel and Carnap [22]) or epistemically (such as Dretske [23]). I propose philosophy look towards understanding semantics as the relationship between a system and its environment, looking for those properties that causally maintain its existence over time, while keeping an eye on avoiding anthropomorphizing such a project, such as erroneously putting top-down constraints on the nature of semantic information having to be propositional or epistemic. This goes back to a debate originally raised by Bar-Hillel and Carnap [22], where they raise the issue that Shannon’s information measure does not answer the question we would expect a complete account of information to answer, namely how semantics arises from the syntax of communication channels. Although there has been much discussion over the years (cf. [22,23,24,37,38]) on how to give a semantic notion of information, many are either too epistemic (such as Floridi’s GDI) or restricted to biological organisms. We can phrase the desideratum as a semantic notion of information which is substrate-neutral, so to speak, one that does not rely on the system in question being biological or having epistemic states of the kind that humans have. For a criticism of Floridi’s semantic notion of information Adriaans [37] argues that Floridi’s conception of semantic information is overly epistemic. Adriaans does a good job of highlighting the two general spheres one might occupy in coming up with a semantic notion of information. On the one hand, one might see information theory as a “handmaiden” to more classical epistemology. Adriaans, however, and I tend to agree with him, sees information theory as a competitor to classic epistemology. I am less certain on how developed a position the latter is, than Adriaans himself appears to be, but I nonetheless see the project of naturalizing consciousness and information as going in the direction Adriaans argues.

Having such a semantic notion of information opens up the possibility that not only biological organisms can have semantic information, but also machines, or something totally alien; since we understand meaning as any information which helps a system to maintain its existence over time. For example, take R2D2, everyone’s favorite beeping robot from Star Wars, there will clearly be information relevant to R2D2 maintaining its existence over time (having the information where the repair and charging stations are) and that will be different in value from that merely syntactic information R2D2 gains from its environment (say it is having the information that the sky is blue, depending on what planet he is on). It is meaningful in the sense of maintaining R2D2′s existence that it has information about its environment concerning where repair stations or charging stations are, as without this relevant information R2D2 would beep no more. That is not to say other information might not have value (more on this in what follows), but it will not be meaningful in the sense that Kolchinsky and Wolpert are concerned with.

Kolchinsky and Wolpert argue that one can give an explanation of how semantics comes from syntax by understanding the relationship between a system and an environment and considering the viability function; between its ability to maintain a state of non-equilibrium and keeping a low state of entropy (e.g., for biological organisms, not dying!). This line of reasoning fits in well also with the free-energy framework advanced by Friston, among others (cf. [39] for a review of this approach to the brain). Any syntactic information which does not figure into maintaining this state of non-equilibrium is ‘meaningless’ in this sense, and thus is not semantic information. Information, however, that does contribute to maintaining this state of non-equilibrium is meaningful, and thus is semantic information. As Kolchinsky and Wolpert explain:

“The **semantic content** of a particular system state x is defined as the conditional distribution (under the optimal intervention) of the environment’s states, given that the system is in state x. The semantic content of x reflects the correlations which are relevant to maintaining the existence of the system, once all other ‘meaningless’ correlations are scrambled away”.

[26]

They are saying that to discover those states of affairs that have meaning one needs to perturb the initial states of system, by way of making interventions on well-defined variables and seeing what information, if any, is lost. If such an intervention affects the optimal viability function, then that contains meaningful information, since its information that is valuable to the system for maintaining its own existence. If an intervention is made and no meaningful information is lost, then all that was scrambled away was the meaningless syntactical correlations. For an overview of how to do perform interventions like this in the context of consciousness science, Baxendale and Mindt [40] argue that a broadly manipulationist interventionist causal framework is the best way to achieve this.

I think motivating this with one of the examples that Kolchinsky and Wolpert use will be helpful at this point, consider the following:

“Consider a distribution over food-catching birds (the system) in the forest (the environment), over a timescale of τ =1 year. Assume that at t = 0 the birds have cached their food and stored the location of the caches in some type of neural memory. If we ‘scramble the information’ by placing birds in random environments, they will not be able to locate their food and be more likely to die, thus decreasing their viability. Thus, a food-catching bird exhibits a high value of information”.

[26]

There is an intuitive level where this makes sense, it is more valuable to a system, or as in the case above an organism, to have information relevant to its continued existence, such as knowing the location of the food cache rather than, say, knowing that the sky is blue; each carry with them a unique set of informational relationships, but one merely indicates a state of affairs (the sky being blue) while the other indicates a valuable state of affairs relevant to the systems continued existence (the cache of food). That is not to say the sky being blue is meaningless, just in this very narrow example. It very well could be the case that in some circumstances this state of affairs could be quite meaningful for the system (more on this later in the section).

The question boils down to what syntactic information is relevant for maintaining a system’s existence. This has to do with discovering the value of the information and this amounts to determining the difference between the viability of the intervened distribution and the actual distribution. What comes out, according to Kolchinsky and Wolpert, is the syntactic information that contributes to maintaining the systems existence. As they put it, “A positive difference means that at least some of the syntactic information between the system and environment plays a causal role in maintaining the system’s existence” [26].

Here, something should be said about how theories of meaning and theories of metasemantics come to bear on the present discussion (thank you to an anonymous reviewer for highlighting the need to discuss this matter). David Lewis [41] writes, speaking of the two questions of how semantic systems gain meaning: “I distinguish two topics: first, the description of possible languages and grammars as abstract semantic systems whereby symbols are associated with aspects of the world; and second, the description of the psychological and sociological facts whereby a particular one of these abstract semantic systems is the one used by a person or population. Only confusion comes of mixing these two topics.” Additionally, although Lewis is right about these two important questions, perhaps the need to distinguish a third question is brought out from our present discussion, one which will hopefully highlight why I have set the issue of various metasemantic theories aside in the present discussion of understanding how semantic information is realized by certain S&D properties/processes. Aside from the questions of how language and grammars as abstract semantic systems latch onto the world, or the psychological/sociological fact of how one of those systems come to be used by a subset of the population; rather, there is a third question, before there was any language (which we use to manipulate and transmit meaning) before any abstract systems of meaning existed, what were the conditions which allowed semantics to form in the first place? Why is there any such thing as meaning, and what are the natural conditions under which the world went from no abstract semantic systems to having meaning? This question, I feel is before the questions Lewis indicates, nonetheless intimately related to the extent that once those basic mechanisms exist, i.e., describing the processes by which meaning and consciousness arise as the result of a certain class of S&D; we then must make sense of how that meaning evolves and iteratively shaped our cognition and mental processes through evolution. In some sense, this means we can place semantic/metasemantic theories to the side momentarily, and focus on the prior question, what are the most basic processes/dynamics/properties that had to be in place for the kind of meaning we utilize daily to be generated?

A further question might arise based on what has just been said and the rest of this section, and that is whether information necessary for survival is more “meaningful” than say abstract information such as mathematical proof, for example? I think this question ignores the initial motivation for the project and is directed at a different level of abstraction than the one this essay is currently directed. We are trying to understand the fundamental processes and dynamics that allow for meaning to arise in the *first place*, the question about how the meaning is used or in what sense something is more meaningful than another is orthogonal to the present discussion, in my opinion. That is not to say this level of analysis is not important, it is just not the present focus of this essay. One might press further, “but didn’t you already say that the blue sky is not necessary for survival and thus meaningless, and are you not making the same error?” That was said in reference to the semantic notion of information given by Kolchinsky and Wolpert, but I am a pluralist about possible conceptions of semantic information, depending on the context. It could be that there are various notions of semantic information which all capture different levels of explanation and are not in conflict with one another. The notion from Kolchinsky and Wolpert is just one possibility, out of presumably many. It could very well be the case that the blueness of the sky could become meaningful for survival, say the sky drastically changed from blue to a deep red, indicating something apocalyptic may be occurring. However, even in this scenario, it is not so much the “blueness” that is meaningful but the change to something drastically different, and it is this surprisal for the system in question that increases the information generated. I am not interested in making the claim that only information directly relevant for survival is meaningful, I merely want to motivate that information relevant for survival was perhaps one of the first steppingstones to meaning being generated in a world previously devoid of meaning. It very well could be, and most likely is, the case that the more we engaged with the world and evolved alongside it we gained more and more abstract semantic contents, as well as the mechanisms to process such abstract information (say in the case of humans a prefrontal cortex). In this sense then, the question of whether something is more “meaningful” than something else lies outside the scope of this present work (thank you to two anonymous reviewers for pointing out the need to comment on this issue).

Some work should be done then on the following question: if systems that causally maintain their own existence overtime can have the kind of intrinsic S&D exemplified by the discussion of Kolchinsky and Wolpert’s work, then is it only living systems that can maintain this kind of trade off discussed in this section? I am hesitant to make the leap that life and consciousness are intimately related. I think that the relationship between the two is superficial, in some sense, in so far as what we prima facie consider “alive” (such as humans) we find consciousness of the kind relevant to ourselves; while also being important, in so far as, living systems are able to maintain their far-from equilibrium state because of the organisms/systems ability to create boundary conditions and isolate its internal workings from the outside world. This ability to isolate internal states from external influence to maintain a “world-inside” so to speak, occurs not only with living organisms/systems, but in consciousness as well. My conscious experience of the world has the kind of boundaries we would expect from a system that has generated borders of the kind necessary to maintain a far-from equilibrium state and to causally maintain its existence over time. Whether based on this we can then equate the way these two processes/dynamics are generated, whether its seemingly plausible, it is nonetheless an inferential step I am not comfortable taking at the present moment. That being said, interesting work to this effect is currently underway, such as Cooke’s Living Mirror Theory of Consciousness [42,43] Friston’s work on free energy framework [39], among others. I am inclined to think that there is an interesting relationship to be explored further between life and consciousness, but I am hesitant to say explaining one of these phenomena requires an explanation of the other or entails an explanation of the other for that matter (thank you to an anonymous reviewer for pointing out the need to elaborate and connect the present discussion to work currently underway to this effect).

There is something important to note, and what is essential in understanding how this discussion relates to the issues in IIT about how meaning is generated and furthermore how this relates to the structure and dynamics argument more generally. I have merely wanted to highlight in this section that it is possible to generate a notion of meaning–a semantic notion of information from purely structural and dynamical properties. However, mind you, this case has been understanding meaning from an extrinsic point of view, that is, some observer intervening on a system (i.e., the bird or R2D2) in the context of its environment (i.e., the location of food caches or repair/charging stations). However, how is the meaning generated by a system *for itself*? This is a slightly different question because now we are asking how is the information presented to the system independent of some observer intervening on the system and environment to determine the difference between the intervened distribution and the actual distribution, how does a system do this on its own? Another way of putting this is how does the bird or R2D2 interface with the relevant meaning that is generated by means of that system maintaining its existence in an environment? The difference between perhaps an organism like the food-caching raven and R2D2 brings another relevant distinction that will be made in the coming sections. Additionally, that has to do with systems being validly interpreted to have semantic value and those that might consciously experience such semantic states. The difference between the raven and R2D2 rests on whether R2D2 is the kind of system that has the sort of internal S&D necessary to realize phenomenal experience. It seems much more likely that it is the kind of feedforward system which may functionally navigate its environment effectively, may undergo the kind of processes necessary to continue maintaining its existence (and be validly interpreted to have semantic value from an external perspective), but nonetheless lacks the right kind of S&D (both intrinsic and internal, discussed in the following sections) to have an experience.

## 5. IIT, Intrinsic Structure and Dynamics, and Approaching the Hard Problem

Integrated Information Theory (IIT) of consciousness is one of the first scientific attempts to give an explanation of phenomenal experience by taking a phenomenology first approach to developing a theory of consciousness. IIT starts by examining what are the essential features of every conceivable experience, or in other words, what are those characteristics of experience that are necessary for every experience; these essential characteristics are formulated as axioms and act as constraints on how to formalize the properties/characteristics of the physical substrate of consciousness (PSC)—these are the physical systems postulates.

Integrated Information Theory begins by reflecting on one’s own phenomenology, that is, it takes as constraints certain indubitable, and what it claims are essential, features of every conceivable experience and translates these into phenomenological axioms, of course, there are those that find this approach contentious (Bayne [44]). From these axioms a set of corresponding postulates are given, meant to explain what the PSC must be like in order to realize the kind of phenomenal characteristics that are essential features of experience. In what follows, I will give a brief overview of the axioms and their translations to physical postulates, to better come to grips with IIT and how it is presented. This will give a launching point for the discussion following, specifically with regard to IIT capturing what I am calling intrinsic structure and dynamics as well as hunting for an intrinsic notion of information.

For our present purposes, an exhaustive overview would be unhelpful since I merely wish to highlight one dimension of IIT’s contribution to the present discussion. I rather am much more interested in looking at IIT from the outside so to speak and how it relates to the present discussion. That being said, I do want to touch on two important aspects of IIT as it relates to the present topic, that is (1) the cause–effect structure (CES) posited by IIT (quantified by Φ^max≥^) and (2) the process and purpose of identifying mechanisms (quantified as φ^Max^) and their relations (to what extent the mechanisms cause–effect repertoires overlap with one another) within the IIT framework. This will be something of an incomplete explanation of IIT for the sake of brevity and focus; if one wants a thorough and exhaustive overview of the contents of IIT, I would recommend reading the papers that most accurately reflect the substantive changes that amount to IIT 4.0 [11,14,27,28,45,46], which present developments and refinements since IIT 3.0 [10]. Despite this, I will endeavor to give all the necessary details as it relates to the overall aim of this essay.

As stated in a recent essay by Haun and Tononi [46]:

“According to integrated information theory, to correspond to an experience, a system in a state must specify a maximally irreducible, specific, compositional, intrinsic cause-effect structure, which is composed of distinctions and their relations. Both distinctions and relations, in turn, must be causal (make a difference) and satisfy intrinsicality (within the system), information (specificity), integration (irreducibility) and exclusion (maximal irreducibility)”.

[46]

As a statement, the above reflects the overall picture in IIT, and each of the above will be gone over now in more detail. Here, I will explain, briefly, the axioms and postulates in tandem in order to make more apparent the meaning behind IIT’s claim that consciousness is a maximally irreducible cause–effect structure. According to IIT consciousness is intrinsic, meaning it is necessarily for itself (following the Cartesian insight that the only thing one cannot doubt is the existence of their own thought/experience), and so according to IIT the PSC must also exist intrinsically for itself. IIT strictly adheres to the Eleatic principle (otherwise known as Alexanders Dictum) that to exist is to have causal power. In the physical sense then, the PSC must have cause–effect power on itself, being able to not only make a difference, but to make a difference upon itself. Consciousness is compositional in the sense that experience is composed of a number of phenomenal distinctions, say the laptop on my desk, the stack of essays surrounding it, and the cup of steaming coffee in my hand, all compose my experience at a given time. The PSC must reflect this compositional nature; thus, the elements of the PSC must have cause–effect power within it, either on their own or in combination with one another. My experience is also specific, in the sense that is composed of a unitary set of phenomenal distinctions that maximally specify my current experience, the particular way it is. The PSC must also reflect this essential phenomenological feature of my experience; thus, it must specify a particular cause–effect structure differentiating itself from all other possible cause–effect structures. These cause–effect structures are a set of causal distinctions and relations specified by all the mechanisms in a system, these mechanisms constrain the probability distribution of past and future states of the system specified by the PSC. Aside from the specificity and compositional nature of one’s experience, it is also unitary, all of the phenomenal distinctions are bound together in such a way as to give one experience as an integrated whole. This integration is reflected in the PSC in the sense that the CES “must be irreducible to the cause–effect structure of specified by non-interdependent subsystems” [14]. Finally, one’s experience is exclusive in the sense that it is definite in its spatial-temporal grain. Additionally, any given time, I am only having an experience of say the laptop in front of me, and not the microscopic elements that compose it or the whole galaxy in which my laptop is contained (it is definite in spatial grain), I am also only ever experiencing this one moment in time, say approximately 40–300 milliseconds, and not the span of pico-seconds or hundreds of years (definite in temporal grain). In this sense then, there is a sort of spatial-temporal sweet spot, or coarse-graining, at which my experience and consciousness occurs. The PSC must also reflect this such that “the cause-effect structure specified… must also be definite… specify[ing] a definite set of cause-effect repertoires over a definite set of elements, neither less nor more, at a definite spatial-temporal grain, neither finer nor coarser” [14]. This constitutes the axioms and their translations into physical systems postulates specifying the way in which the PSC must exist in order to satisfy all essential features of experience. Of course, such a list is constantly being refined and made more precise and, in some sense, are neither set in stone nor perfectly captured in common language. Nonetheless, the axioms and postulates provide enough specificity and constraint to then begin testing and refining the measure of integrated information in a system.

If a physical substrate satisfies all the above postulates than a system can be said to satisfy the central explanatory identity of IIT:

“An experience is identical to a [cause–effect structure], meaning that every property of the experience must correspond to a property of the [cause–effect structure] and vice versa. Note that the postulated identity is between an experience and the [cause–effect structure] specified by the PSC, not between an experience and the set of elements in a state constituting the PSC. The quality or content of consciousness–which particular way the system exists for itself–corresponds to the form of the [cause–effect structure]. The quantity of consciousness–how much the system exists for itself–corresponds to its irreducibility φ^Max^”. [14] (The quote has been modified to reflect IIT’s most recent exposition changing “conceptual structure” to [cause–effect structure] in order to make the exposition clearer. The same has been done with the change of “concept” to “distinction” throughout the quotes of IIT.)

To go some ways in unpacking what all this means: A cause–effect structure determines the particular way the PSC specifies an experience that satisfies the five axioms of intrinsicality, composition, information, integration, and exclusion. This cause–effect structure is composed of mechanisms and relations. Mechanisms for IIT, are unique cause–effect repertoires specified by elements, or collection of elements (higher-order mechanisms), which determine the unique contents they specify (distinctions). A favorite example being the hypothetical mechanism in the fusiform gyrus that fires when looking at the face of Jennifer Aniston. This mechanism (say neuron/neuronal group) maximally specifies this content in the overall cause–effect structure (composed of mechanisms and their relations). The meaning being intrinsic to the system, and in an important sense, meaning is phenomenally constituted according to IIT, “the meaning is the feeling” (to borrow Giulio Tononi’s phrasing for this.) so to speak. This is relevant since we are concerned with qualitatively distinct structural and dynamical properties of certain systems, and if IIT is correct, then there are systems organized in such a way (say the neural architecture of the human brain) that expresses qualitatively different S&D, formally characterized by integrated information. Given that a system, according to IIT, that has a non-zero level of integrated information can only be properly characterized by “unfolding” its cause–effect structure, into its mechanisms, distinctions, and relations to fully appreciate the causal power of the system it would be a mistake to merely characterize it extrinsically, i.e., as just consisting of the kind of structural and dynamical features detailed by Chalmers.

One way to understand why this would be a mistake, is to look at the recent unfolding argument given by Doerig et al. [47] which argues that causal structuralist theories of consciousness, such as IIT, fail to specify what systems are conscious because any recurrent network can be implemented on a feedforward network and preserve the functional states/outputs of the system. Since they also claim that the only way to do a science of consciousness is functionally (agreeing with Cohen and Dennett’s [48] view that functional explanation is the only kind of explanation for a science of consciousness). For a view contrary to this position, in Ellia et al. [49] we argue that the functionalist zeitgeist of the past decades is woefully inadequate to capture phenomenology, despite its many success in explaining other aspects of mentality. What the unfolding argument fails to appreciate is exactly the point of this essay in understanding the nuances in S&D. The reason being, that merely modeling or describing a systems extrinsic S&D such that merely functional states are preserved misses the point entirely, because equivalent models do not mean equivalent internal/intrinsic states of the system. If one thinks that extrinsic characterizations that are equivalent mean equivalent internal states, then there is little to dissuade that there is something relevant about trying to capture a systems intrinsic/internal S&D. My hope is that I have at least given enough reason to doubt that extrinsically characterizing a system leads to capturing its intrinsic properties. If one accepts the hard problem and wants a S&D explanation of consciousness, one should drop this faulty assumption and accept that extrinsic or functional equivalence is a faulty guide (despite functional explanation being useful in other instances) when it comes to intrinsic/internal equivalence.

The method discussed in Section 4 is similar (though different in relevant ways) to that method by which IIT determines the optimal intervention for quantifying the level of φ^Max^ of a mechanism in a system. IIT looks to find those causal/informational relationships that form irreducible sets, that is elements that form an integrated whole, where the loss of some element affects the amount of information the group of elements taken together express (integrated information). The most recent formal expression of this value can be found in Barbosa et al. [27] and Barbosa et al. [28] the unique distance measure developed therein will be the formalism used for the updated IIT 4.0 version of the theory currently in development. The idea being, that finding such irreducible sets reveals how the current state of the system shows you what states they were in in the past and how they constrain future states of the system, this is given by the transition probability matrix (TPM) of all the units in the candidate system at a time. The way of determining this is placing a set of elements in a state and performing ‘cuts’ (or directed interventions on specific variables, by say introducing noise into one of the elements, etc.) and seeing how the whole is affected by the intervened variable. If the amount of integrated information that the elements express is less once the target variable has been intervened on, then that set of elements forms a collection of elements (mechanism) that has integrated information. As it is put in IIT 3.0, “[e]ach [distinction] of a mechanism in a state is thus endowed with a maximally irreducible cause-effect repertoire (MICE), which specifies what the [distinction] is about (its quale “*sensu stricto*”), and its particular φ^Max^ value, which quantifies its amount of integration or irreducibility” [10]. That is to say, that each mechanism that specifies a distinction has a meaning identified by only that mechanism and recall that it has this because of its unique cause–effect repertoire that it specifies in the system as a whole.

One important difference here between the approach from Kolchinsky and Wolpert and that of IIT is that IIT is concerned with not only the meaning that can be extrinsically interpreted from the outside (i.e., through interventions on a target system), but rather what the meaning is that is intrinsically brought to the fore to the system; how do these unique informational/causal relationships constrain the past and future states of a system as it evolves dynamically over time? I want to tread carefully here, as some might think I have assumed the truth of the views discussed thus far. It is best to read any assumptions regarding these views as a conditional, merely to help facilitate the discussion of the kind of S&D properties I want to highlight in this work. It may be the case that the particularities of the works discussed may be off, and their use in this context is meant to be illustrative (thank you to a reviewer for pointing out the need for this clarification). There is, however, an important connection between these two methodologies for providing a semantic notion of information by discriminating between purely syntactic information and information that causally constrains the system to facilitate its continued existence. IIT is just asking the further question, which is, what makes a system that has *internal **and** intrinsic* meaning different from a system that has purely extrinsic meaning? According to IIT, it is when a system has the right kind of intrinsic cause–effect power. The following section will delve more deeply into this distinction and give an argument as to why not all structure and dynamics are equal.

## 6. Not All Structure and Dynamics Are Equal

We have seen thus far that there are a number of different kinds of structure and dynamics. There are those extrinsic structural and dynamical properties which merely indicate a system’s syntactical features those features which Chalmers calls structural and dynamical in his characterization of what physical explanation tells us. However, as Kolchinsky and Wolpert show, not all syntactical information is “meaningless” so to speak, and thus we can distinguish information that is meaningful to a system from that which is merely a byproduct of the connection between a system and its environment. In terms of meaning, we can consider all other information not directly related to a system’s continued existence as ‘noise’ at this level of abstraction. We already see here that there is a difference between intrinsic S&D and extrinsic S&D. However, there is a further level at which not all intrinsic structural and dynamical features of a system are the same, depending on what perspective one takes with regard to the target system.

I now want to make a further distinction, between External S&D and Internal S&D.

**External S&D**: those meaningful or meaningless S&D properties of a system, observed from an external perspective. Those which are meaningful are necessary for that system to causally maintain its own existence as an observable entity with certain behaviors. Those which are meaningless are not necessary for the system to maintain its existence overtime.

**Internal S&D**: those meaningful S&D properties of a system that are necessary for that system to causally maintain its own ***intrinsic*** existence; that is, to maintain an existence which has an ***internal perspective*** through its own experience.

Some may think this a rather odd distinction to make but in what follows, I will endeavor to explain why such a distinction is not only useful, but necessary, for understanding that not all structural and dynamical properties of a system are equal and why IIT’s account is so informative on the matter as it concerns consciousness.

It would be useful to quickly recap the various kinds of structural and dynamical properties that have so far been discussed. Initially (Section 1) we discussed structure and dynamics as Chalmers conceives of them as a facet of his hard problem of consciousness, this type of S&D is what I am calling extrinsic S&D (a non-meaningful variant of external S&D). Next (Section 2) we discussed some recent work in complexity science in developing a semantic notion of information which I argued reveals that there are not only structural and dynamical features of the kind Chalmers describes, but also intrinsic S&D (as described by Kolchinsky and Wolpert [26]) of the kind we get when we look at the interaction of elements in the system, rather than the elements themselves (this would be the meaningful variant of external S&D, intrinsic but also interpreted from an external perspective).

What about the situation in which we are not only concerned with whether a system can be validly *interpreted* to contain meaning, but there is *something it is like for* that system to have such meaning, as is the case in phenomenal experience? This is why, I think, it is important to understand that not only are extrinsic and intrinsic structure and dynamics not equal, but there is also a further level of description contained within intrinsic structure and dynamics. That is there is intrinsic structure and dynamics one might characterize about a feature of reality as it relates to externally capturing what processes maintain a systems existence over time (what IIT calls extrinsic existence, for example) those that are external to a system (i.e., an observer intervening on some variable and determining the semantic value of some set of data from comparing the distributions). Then, there are those intrinsic structural and dynamical features of a system which relate to capturing what processes maintain a system’s existence over time *internally* to said system (i.e., my consciousness of the semantic features of myself taken as the system in question, what it is like for me to experience the world).

External and internal S&D are merely the different perspectives one can take on a system or as a system on its processing (or more accurately has the ability to take), that is, I can be observed as having certain extrinsic S&D features, say the molecules that compose my body or my motion through a room being tracked by cognitive scientists for some experiment. These would amount to being treated as an object of inquiry, in the third-person sense, from an external perspective. Some systems examined from such an external perspective may exhibit certain intrinsic S&D of the kind discussed in Section 3, revealing that there is meaning associated with such processes, such as the food-catching bird or the beeping R2D2. Others, more commonly, will exhibit merely the kind of extrinsic S&D features which Chalmers rightly points out will fail to account for the qualitative aspects of our brains physiological processes. Take for example the behavior of a gas in a vacuum, there is no “meaning” to be found in that system reaching a state of equilibrium, there is merely the indication of various syntactical states of affairs, there is no relevant sense in which those processes are differentiable enough to be relevant for that system to maintain its existence in the sense we are concerned with, from the intrinsic perspective. As concerns the latter extrinsic features, Chalmers is most assuredly correct to think such an explanation would never account for the felt aspect of experience, the what-it-is-likeness, but if our present discussion has been successful, this should be wholly unsurprising and uninteresting. These are both external S&D of a certain variety, the former more interesting for questions about the mind, as they reveal a system that may have semantic properties. The former being more relevant for understanding the causal structure of the world, one which is revealed to us by the typical tools of scientific inquiry. What is interesting for the question of how phenomenal experience arises as the result of certain structural and dynamical properties is not whether I can be observed to have semantic properties, but that the system in question (i.e., myself) experiences those meaningful properties–whether there is something it is like for me to undergo the causal processes necessary to maintain my own existence over time.

I am the type of system (and if the reader is similar enough to myself, will also be the type of system) which experiences the transitions intrinsically of myself from one state to another, i.e., what it is like to experience the world. Assuming that the only kind of structure and dynamics are extrinsic S&D is a sure-fire way to miss out on the question worth asking, which is, if we find ourselves in a world governed by a complicated array of structural and dynamical properties what are the ones which have an internal perspective on their own processes as they unfold? If we are looking for a natural explanation of phenomenal experience that should be what our concern is and shedding light on the nuance of what kinds of structural and dynamical properties/processes there are, is the right way to advance this effort.

I argued previously [16] that if we take the hard problem and the structure and dynamics argument at face value, and accept them conditionally, then IIT fails to overcome the hard problem because it provides a purely structural and dynamical notion (that is as Chalmers conceives of S&D) of information. What we have seen thus far is that *not all structure and dynamics are equal*, and that there are relevant differences in structural and dynamical features of certain systems that should and need to be investigated further. If Chalmers’s structure and dynamics argument is that there could not possibly be a S&D explanation of phenomenal experience, given how he defines structure and dynamics, then his argument does not apply to structure and dynamics that fall outside the scope of his definition. I have shown that there is nuance in structure and dynamics, even in the external case as was shown in Section 3, between meaningful and meaningless syntactical information. The further task is to take those lessons and apply them to the question of those systems which can be said to have semantic information, do those same systems have an internal perspective, is there something it is like for those systems to experience the meaningful causal states that maintain that systems survival over time? Additionally, if so, what are the relevant S&D differences between those systems to warrant such an internal perspective? Both of these questions are susceptible to natural investigation, given we have the correct conceptual framework and tools to perform such an investigation. The project should be to develop and discover those, not to preclude the possibility from the outset by a restricted characterization of structure and dynamics. Merely showing that not all structure and dynamics are equal is enough to resist the claims of the structure and dynamics argument, and without that argument, there seems little reason to accept the hard problem as the insurmountable obstacle it appears to be.

Before we finish on this note, it would be beneficial to briefly clarify, for the sake of interpretation, what the goal of this work has been. I do not so much want to argue that accepting IIT, or that any system with non-zero phi, is the only way of explicating what it would mean to capture the relevant properties of certain systems that have internal S&D. Rather, I think IIT is the most promising way of doing this at this early stage. I think it is outside my epistemic ability to make such a claim. Rather, if we are to find a path forward, IIT is currently one of the best places to start. However, I am something of a pluralist about possible explanations and would be delighted if other theories/frameworks arose that were able to capture the kind of S&D properties I have tried to outline in this essay. This essay though is merely a first step, a crack in the door and much more needs to be done and said in order to fully argue for this point. It should just be clear that IIT is one possibility, perhaps only currently, but that does not preclude the possibilities of other theories doing so in the future (thank you to an anonymous reviewer for pointing out the need to make this clarification of what I am saying).

I have endeavored to show that not all structure and dynamics are equal, or at the very least, it appears there may be relevant differences in structural and dynamical properties of certain systems that are not captured by Chalmers’ description of structure and dynamics. If there are S&D properties that are not captured by Chalmers’ description and definition of what is structural and dynamical then the structure and dynamics argument is only true for those properties that are captured by his description. Intrinsic (both external and internal) structural and dynamical properties fall outside the scope of Chalmers description and definition, and thus fall outside the scope of his structure and dynamics argument. If this is so, then perhaps there are structural and dynamical explanations of a certain variety that may account for phenomenal experience. I merely want to show, in this essay, that not all structure and dynamics are equal and if this is so, then Chalmers structure and dynamics argument may not exclude a certain variety of structural and dynamical explanation of consciousness. Rather, what Chalmers’ argument would show is that one cannot give, what I have called in this essay, an *extrinsic/external structural and dynamical* explanation. That leaves open the possibility of exploring a specific class of S&D properties, *intrinsic/internal structural and dynamical properties*, and how those might explain consciousness and phenomenal experience. Specifically, those properties of a system I am calling *internal structural and dynamical* properties. The following section (§5) will briefly look at some of the implications this has for the metaphysics of consciousness.

## 7. Metaphysical Implications

The hope thus far has been that I have at least given reason to think that not all structure and dynamics are equal, if this is so, then what are the implications for those arguments against physicalism more generally that purport to show an epistemic and ontological gap between structural and dynamical features (treated as physical features) and the mental (phenomenal experience)? I do not wish to vindicate physicalism or argue in favor of anti-physicalism; I merely want to highlight that given our more nuanced picture of structural and dynamical features we now have access to we might be able to close the gap with such a picture of S&D. Whether that means one should be a physicalist or anti-physicalist I will leave that to the judgement of the reader. I will, however, in this brief section give my own take on the implications of not all S&D being equal has on the metaphysics of consciousness. I will present two horns of interpretation this might lead one to accept: that it vindicates physicalism or that it damns physicalism even further.

If the physicalist accepts an expanded notion of structure and dynamics to accommodate the differences I have endeavored to highlight in this essay, then it seems physicalism can shake the shackles of the hard problem. This, however, would require the physicalist to admit that systems are not fully characterizable using the traditional tools and concepts of the physical sciences (for an argument why this is not problematic when it comes to consciousness science, cf. [49]), creaking the door for the fuzzy concept of intrinsicality (something I suspect a good deal of physicalists would resist). If so, then physicalism seems just as doomed as always, since Chalmers structure and dynamics argument (and thus the hard problem) holds with a purely extrinsic conception of structure and dynamics. That being said, perhaps many physicalists will be unfazed by this, since it may well not jar with their intended metaphysical aspirations in terms of accommodating consciousness into the natural order of the world. In some sense, perhaps this vindication is only targeting those physicalists who feel the sting of the hard problem/structure and dynamics argument and wish to rid themselves of such a sore spot while retaining their physicalism.

On the other hand, and what I see as the more likely result, is that what I have endeavored to show actually damns physicalism further, showing that its narrow conception of the natural world leaves too much wanting in terms of a complete explanation, especially in terms of consciousness and its place in nature. This would be tantamount to accepting that Chalmers characterization of physicalism and structure and dynamics is accurate, and thus because it fails to accommodate an expanded notion of structure and dynamics leaves out any possibility of explaining phenomenal experience. That does not by any means mean that the project of monism is foregone or that we all have to end up being dualists, such as Chalmers himself, but rather a monism that accommodates an expanded conception of structure and dynamics should be developed.

Personally, I think that the only monism that shows any hope of accommodating consciousness into the natural order of the world is a form of neutral monism [5,50]. Of course, the specifics are what matter and unfortunately a promissory note is all that is possible at this early stage, though I have attempted elsewhere to begin developing a form of information-theoretic neutral monism [51] which attempts to integrate some of the insights of this present work.

I have endeavored in this present work to offer an explanation of how a theory might give a S&D explanation of certain variety about consciousness, which dissolves the S&D argument of Chalmers. My hope is to vindicate the process of developing a naturalized explanation of consciousness from the hard problem and the structure and dynamics argument, and by extension, those gap arguments that have arisen as a result. I stay agnostic on whether this vindicates physicalism as a result, I merely wish to vindicate structural and dynamical explanation of a certain variety. The project of then developing a metaphysics/ontology of consciousness that accommodates this picture of S&D is one I am currently undertaking.

## 8. Conclusions

It has been the aim of this essay to show that not all structure and dynamics are equal, as a way of opening up the possibility of looking for the right kind of S&D explanation of phenomenal experience. I have argued that we should look towards IIT, or some similar view, which might capture what I am calling *internal S&D* as a fruitful way forward in explaining phenomenal experience. Opening this possibility I think represents a significant step forward in what kind of explanations would suffice to explain phenomenal experience and will hopefully show the main flaw behind the hard problem of consciousness. Although I share sympathies with Chalmers on this matter, I think ultimately his characterization of S&D fails to take into account the interesting and illuminating processes and relationships that arise when we focus on intrinsic interactions rather than the extrinsic components of certain systems.

## Data Availability

Not applicable.
